# Teamwork makes the dream work: functional collaborations between families, scientists, and healthcare providers to drive progress in the treatment of Leigh Syndrome

**DOI:** 10.1186/s13023-023-02871-7

**Published:** 2023-11-16

**Authors:** Jesse D. Moreira, Karan K. Smith, Sophia Zilber, Kasey Woleben, Jessica L. Fetterman

**Affiliations:** 1 Evans Department of Medicine, Whitaker Cardiovascular Institute, Boston University Chobanian and Avedisian School of Medicine, 02118 Boston, MA USA; 2 Cure Mito Foundation, 6808 Old Glory Ct., 75071 McKinney, TX USA

**Keywords:** Leigh syndrome, Mitochondria, Mitochondrial genetics, Mitochondrial disease, Community, Patient registries, Symposium

## Abstract

**Background:**

Leigh syndrome, an inherited neurometabolic disorder, is estimated to be the most common pediatric manifestation of mitochondrial disease. No treatments are currently available for Leigh syndrome due to many hurdles in drug discovery efforts. Leigh syndrome causal variants span over 110 different genes and likely lead to both unique and shared biochemical alterations, often resulting in overlapping phenotypic features. The mechanisms by which pathogenic variants in mitochondrial genes alter cellular phenotype to promote disease remain poorly understood. The rarity of cases of specific causal variants creates barriers to drug discovery and adequately sized clinical trials.

**Body:**

To address the current challenges in drug discovery and facilitate communication between researchers, healthcare providers, patients, and families, the Boston University integrative Cardiovascular Metabolism and Pathophysiology (iCAMP) Lab and Cure Mito Foundation hosted a Leigh Syndrome Symposium. This symposium brought together expert scientists and providers to highlight the current successes in drug discovery and novel models of mitochondrial disease, and to connect patients to providers and scientists to foster community and communication.

**Conclusion:**

In this symposium review, we describe the research presented, the hurdles ahead, and strategies to better connect the Leigh syndrome community members to advance treatments for Leigh syndrome.

## Introduction

Leigh syndrome is an inherited neurometabolic disorder. Leigh syndrome is estimated to be the most common form of mitochondrial disease, affecting children with a prevalence of ~1 in 40,000 births [3, 4]. Leigh syndrome was first described in 1951 by the physician Archibald Denis Leigh [1]. In the 72 years since then, we have made tremendous progress in identifying causal pathogenic variants, which span over 110 different genes in both the mitochondrial and nuclear genomes [2–4]. Despite the discovery of the underlying pathogenic variants that cause Leigh syndrome, little progress has been made in the treatments available.

Owing to the wide array of causal variants across many genes identified in Leigh syndrome, the cellular mechanisms driving disease progression are thought to be heterogeneous [2, 5]. Different aspects of mitochondrial function are likely altered by the variants in different genes, creating complexity in the biochemical defects and consequently, challenges in identifying drug targets effective across all patients [6, 7]. The prognosis for Leigh syndrome is generally poor, with mortality rates of nearly 80% by age 20 [6]. No Food and Drug Administration (FDA)-approved therapies for the management of Leigh syndrome are available.

The Leigh syndrome research community, and the mitochondrial disease research field in general, faces significant barriers to the discovery of novel treatment strategies. Due to the rarity of the disease and heterogeneity in pathogenic variants, it is difficult to recruit patients for adequately sized clinical studies, as patients are geographically dispersed. Limitations in the models available for studying mitochondrial diseases create barriers to drug discovery and as a result, the underlying mechanisms of Leigh syndrome and specific causal variants are poorly understood.

As such, the Boston University integrative Cardiac Metabolism and Pathophysiology Program (iCAMP) and the Cure Mito Foundation co-hosted a virtual symposium entitled “Empower & Inspire: Understanding and Accelerating Research for Leigh Syndrome” to bring together healthcare providers, scientists, and families to problem solve together. The symposium started with an introduction from Kasey Woleben who described the history of the Cure Mito Foundation and highlighted the importance of the families coming together. The introduction was followed by a keynote talk by Dr. Vamsi Mootha who ended by sharing that this is an incredibly promising time for Leigh syndrome research but underscored that real progress will require all stakeholders coming together collaboratively. Dr. Saima Kayani then provided a primer and background on the disease in her talk titled, “Leigh Syndrome 101.” Her talk primed the attendees for the rest of the symposium, by covering disease etiology, clinical presentation, natural history and disease progression, and current standard of care for Leigh syndrome.

All speakers and presentation titles are provided in Table 1 and the link to the free and publicly available video recordings of the presentations is also provided. Most presenters discussed published findings and relevant citations are included throughout the text. It should be noted that some of the data that was presented is unpublished and as such, has not been peer-reviewed, which is indicated when relevant. Competing interests are noted in the case of industry-funded studies. Collectively, we discuss here the advances presented at the symposium. We highlight novel disease models, therapeutics in the pipeline, patient recruitment and database strategies, and family needs to bring together the community to accelerate research into this devastating disease.

**Table 1:** Speakers at the Leigh Syndrome Symposium

Speaker	Affiliation	Title of Presentation
Kasey Woleben	Cure Mito Foundation	Introduction
Vamsi K. Mootha, MD	Harvard Medical School, Broad Institute of Harvard and MIT	Keynote: ​Advances in Leigh Syndrome Research: Sterling Past, Golden Future
Saima Kayani, MD	University of Texas Southwestern Medical Center	Leigh Syndrome 101
Steven J. Gray, PhD	University of Texas Southwestern Medical Center	General Overview of Nervous System Targeted Gene Therapy
Michal Minczuk, PhD	University of Cambridge, Pretzel Therapeutics	Mitochondrial Genome Engineering In Vivo
Jessica L. Fetterman, PhD	iCAMP lab, Boston University School of Medicine	Gaining Insights into Mitochondrial Diseases Using Stem Cells
Alessandro Prigione, MD, PhD	University of Düsseldorf, Germany	Patient-Derived Stem Cells and Brain Organoids for Modeling and Drug Discovery of Leigh Syndrome
Simon C. Johnson, PhD	University of Washington, Seattle Children’s Research Institute	Immune-Mediated Disease Pathogenesis in Leigh Syndrome
Joseph J. Bellucci, PhD	Rarebase	Applying A Precision Medicine Drug Discovery Platform to SURF1 Leigh Syndrome
Anne N. Murphy, PhD	Cytokinetics	Challenges in Drug Discovery for Mitochondrial Disorders
Matthew B. Klein, MD, MS, FACS	PTC Therapeutics	Insights from over a Decade of Experience in Mitochondrial Disease Drug Development
Volkmar Weissig, ScD, PhD	Midwestern University	Development of New Drugs for Mitochondrial Diseases with a Focus on Leigh Syndrome
Sophia Zilber	Cure Mito Foundation	Leigh Syndrome Global Patient Registry Updates
Rhonda Facile, MS	Partnerships and Development, CDISC	CDISC and How Data Standards Can Help Drive Development of Mito Treatments
Parag Shiralkar, MS, MBA	Sumptuous Data Sciences	Interoperability of Leigh Syndrome Patient Registry Data with Regulatory Submission Standards
Alexandre Bétourné, PhD, PharmD, PMP	Critical Path Institute	The Rare Diseases Cures Accelerator Data and Analytics Platform: Accelerating Drug Development for Rare Mitochondrial Disorders.
Shannon Rego O’Rourke, MS, CGC	Allstripes	SURF1-related Leigh Syndrome Health Data Insights
Parents of Patients with Leigh Syndrome	N/A	Parent panel Jennifer Linnebach (USA) Krisztina Ferencz (Romania) Jorgelina Alvarez Barral (Argentina)
Rachel Kramer, PhD	Clinical Psychologist, Private Practice	Parenting Children with Mitochondrial Disease: Finding Space for Parental Well-Being
Jessica L. Fetterman, PhD and Kasey Woleben	iCAMP lab, Boston University School of Medicine and Cure Mito Foundation	Closing Remarks

The sessions were moderated by Kasey Woleben (Cure Mito), Ethan Perlstein, PhD (Perlara), Kevin Freiert (Salem Oaks, Cure Mito), Danielle Boyce, MPH, DPA (John Hopkins School of Medicine, Cure Mito), Liz Morris (parent and caregiver collaborator).

### Recent research in Leigh Syndrome and mitochondrial disease mechanisms

Several advances in research on Leigh syndrome were highlighted during the symposium, specifically those involving gene editing techniques and the use of induced pluripotent stem cells (iPSCs). Notably, currently available gene editing tools, particularly Clustered, Regularly Interspaced Short Palindromic Repeats (CRISPR)-Cas techniques, work well for editing the nuclear genome but lack efficiency in mitochondrial genome editing [8]. Speakers emphasized the utility of gene therapy as a tool to shift heteroplasmy levels and correct pathogenic mitochondrial deoxyribonucleic acid (DNA) variants, but such therapeutic strategies are still years away from implementation in the clinic. As a result of the inefficiencies of CRISPR-Cas techniques, Dr. Michal Minczuk and his team have engineered nucleases, including mitochondrial zinc fingers, which allow cleavage at specific pathogenic mitochondrial DNA sequences [9]. The use of mitochondrial zinc fingers in mouse models has successfully corrected mitochondrial DNA variants in vivo and improved measures of mitochondrial function in the heart [10].

Dr. Steven Gray provided an overview of adeno-associated viruses as a vector for gene therapy [11]. He presented data showing that adeno-associated virus 9-mediated expression of human *SURF1* in *Surf1* knockout mice restored *Surf1* expression and normalized measures of mitochondrial dysfunction, including complex IV levels and lactic acidosis [11]. However, the value of the *Surf1* knockout mouse model has been contested as not reflective of the symptomology observed in patients; future studies are needed to evaluate the efficacy of adeno-associated virus 9-mediated expression of the genes affected in other models of Leigh syndrome. While preclinical studies and some human clinical trial data are promising, research is still needed to support the utility of adeno-associated viruses for gene delivery in humans, as current treatment with adeno-associated viruses have resulted in mixed efficacies across non-mitochondrial disease applications. Further, adeno-associated viruses for gene delivery are limited to a single dosing event due to immune reactions, which means that patients would likely require frequent injections. Adeno-associated virus-based clinical trials have not yet been performed for the treatment of Leigh syndrome but may represent a potential avenue for treatment upon further exploration.

Dr. Joseph Bellucci of Rarebase discussed the company’s platform for mechanistic studies of rare diseases. He presented unpublished data illustrating the utility of transcriptomics as an outcome for evaluating drug efficacy using iPSC-derived neurons harboring a *SURF1* variant. Such an approach has the potential to identify multiple therapeutic options in a single screen. Further, the use of iPSC-derived neurons allows for personalized medicine approaches in drug screening as iPSCs can be readily generated from a blood sample provided by patients.

In addition to gene editing, new cell models are being developed to study mitochondrial disease. Dr. Jessica Fetterman presented on the promise of human iPSC-derived cardiomyocytes for advancing our understanding of the cardiac involvement in mitochondrial disease [8]. iPSCs derived from donated blood or skin samples from patients with Leigh syndrome provide the ability to study mitochondrial disease in clinically relevant cell types as the cells contain the genetic background of the patient and can be differentiated into any cell type. The use of iPSCs will likely transform our understanding of the mechanisms underlying differences in treatment response between patients and presents the opportunity to create personalized medicine approaches for the treatment of mitochondrial disease.

Dr. Alessandro Prigione demonstrated the utility of using human-derived brain organoids for drug screening [12]. Dr. Prigione’s group performed drug screens using iPSC-derived neuronal progenitor cells from two patients with Leigh syndrome harboring *SURF1* pathogenic variants [12]. The application of patient-derived iPSCs for large-scale drug screening shows promise. Dr. Prigione proposes a platform whereby a group would first use iPSC-derived cells to screen potential therapies. Then, compassionate release applications of promising therapeutics may be possible to test safety and efficacy in clinical applications in small numbers of patients.

Dr. Simon Johnson discussed his lab’s work on the pathogenesis of Leigh syndrome. In particular, he discussed the involvement of immune-mediated inflammation in central nervous system lesions in the *Ndufs4* knockout mouse, a model of Leigh syndrome [13]. Treatment with a Colony Stimulating Factor − 1 Receptor inhibitor attenuated disease progression in the *Ndufs4* knockout mice, likely by reducing the neuroinflammatory-mediated lesion progression, and may represent a novel therapeutic target [13].

Dr. Vamsi Mootha presented a pre-clinical therapeutic strategy for the treatment of Leigh syndrome – the use of hypoxia. In addition to other mechanisms, the presence of unused oxygen when mitochondrial respiration is disrupted in mitochondrial disease can result in damage to cells through free radical generation. Thus, lowering oxygen tensions in mitochondrial patients may relieve cellular oxidative stress and decrease downstream cellular damage. Dr. Mootha’s group showed the benefit of hypoxia in alleviating multiple signs of disease, including motor dysfunction and body temperature dysregulation in a mouse model of Leigh syndrome [14].

### Clinical trials and advances in mitochondrial therapeutics

As outlined by Dr. Anne Murphy, clinical trials and drug development in mitochondrial disease are more challenging compared to drug development for other diseases. Mitochondrial diseases are the result of genetic variants in either the mitochondrial or nuclear genome. Multiple genes are implicated in mitochondrial diseases, spanning different functions within the mitochondrion. The locus heterogeneity likely results in different biochemical abnormalities even within a single mitochondrial disease, including Leigh syndrome. This complicates efforts in discovering drug targets and therapies that are effective broadly across patients despite differences in their biochemical abnormalities. Additionally, since metabolic pathways are tightly interwoven through shared substrates and cofactors, multiple drugs may be needed to effectively circumvent or restore the disrupted metabolic pathways. Dr. Murphy also highlighted that having to navigate two genomes that talk to one another through relatively unknown mechanisms is quite difficult. Lastly, the mitochondrial proteome has also only recently begun to be sufficiently annotated for the construction of mitochondrial functional pathways, through efforts such as MitoCarta 3.0 from Dr. Vamsi Mootha’s group [15].

Compared to monogenic diseases, mitochondrial diseases, and Leigh syndrome in particular, are caused by many different genetic variants located across hundreds of genes. The phenotypes of patients with Leigh syndrome are similar; however, while some of the biochemical pathways involved in the pathogenesis of Leigh syndrome may be shared across all affected genes, some of the pathways involved may also differ, likely contributing to the heterogeneity in responses to novel therapies. As Leigh syndrome is a rare disease, this symposium highlighted the need to recruit individuals with different disease-causing variants into the same trials, sometimes known as “lumping.” Leigh syndrome clinical trials are likely to have mixed outcomes as only some patients will likely benefit based on whether the therapy effectively targets their variant/gene-specific disease mechanism. The differential responses to the therapeutic may require stratification of outcomes by gene or protein affected in a given patient cohort to identify the patients who derive benefit from the therapy. Moreover, in contrast to lumping when disease mechanisms are similar in trial recruitment, “splitting” by different disease mechanisms may also be necessary to identify the patients with the greatest benefit of novel therapeutics.

While there are certainly challenges in developing novel mitochondrial therapeutics, the symposium highlighted several examples of drugs in the pipeline. Those drugs presented in the symposium for mitochondrial disease trials are in the class of reactive oxygen species scavengers. Such drugs are designed with the intent of preventing excessive levels of reactive oxygen species from damaging the cellular components. In the symposium, Dr. Volkmar Weissig led a discussion on how drugs like Vatiquinone and Sonlicromanol have shown benefit in small clinical trials of patients with mitochondrial disease. In a study of 13 children and 1 adult with mitochondrial disease at risk of progressing to end-of-life care within 90 days, Vatiquinone treatment resulted in clinical improvement in 11 patients, of whom 3 partially relapsed [16]. In another study, Vatiquinone given to 5 patients with Leber’s Hereditary Optic Neuropathy, a mitochondrial disease resulting in vision loss, resulted in arrest of disease progression and reversal in 4 of 5 patients, with 2 patients having complete recovery of visual acuity [17]. While these data show promise, larger-scale, randomized placebo-controlled clinical trials are warranted, which will likely be facilitated by patient registries. Larger and longer trials may shed more light on whether reactive oxygen species scavengers are beneficial for the treatment of only certain clinical manifestations of mitochondrial disease, or whether the improvements demonstrated were merely the result of the natural ebbs and flows in disease progression.

### Patient registries, data collection, and building a digital community

The absence of patient registries and harmonized data on Leigh syndrome disease progression is a major hindrance to the development of treatments. Dr. Matthew Klein echoed these sentiments in a summary of insights from his own experience in drug development for mitochondrial diseases. As patients are located in geographically distant places, performing centralized clinical trials remains difficult. Registries allow healthcare providers and scientists to locate and enroll patients in multi-site trials to ensure accessibility to all in need and to enroll the highest number of patients possible. Moreover, data collection from many patients and data harmonization efforts allows more patients to be enrolled by enabling clear inclusion and exclusion criteria. In response to this issue, the Cure Mito Foundation, in collaboration with Sanford Health, has formed a global Leigh syndrome patient registry, already successfully registering over 200 patients to date. Multiple speakers, including Drs. Murphy and Mootha, emphasized the importance of the registry in the drug development process.

Sophia Zilber gave an overview of the Cure Mito registry design, accomplishments thus far, and future directions. She emphasized the benefits of a patient registry, including facilitation of clinical trial recruitment, providing data on the prevalence and life history of the international Leigh syndrome population, and most importantly, ensuring the data and results are accessible to researchers and the patient community to advance our understanding of the disease. The Cure Mito Leigh syndrome registry has partnered with Sumptuous Data Sciences to ensure that their data collection meets the standards of the Clinical Data Interchange Standards Consortium (CDISC), required by the FDA and other regulatory agencies. Parag Shiralkar from Sumptuous Data Sciences explained the technical aspects of transforming collected data to meet CDISC standards. The vice president of CDISC, Rhonda Facile, provided an overview of CDISC and highlighted how harmonized data collection can improve data analysis, aggregation, and sharing.

We also learned about Cure Mito’s partnership with the Critical Path Institute (C-path), a nonprofit organization that creates a neutral environment for those in industry, academia, and other stakeholders in the drug development process to form public-private partnerships. Dr. Alexandre Bétourné provided an overview of Critical Path Institute’s Rare Diseases Cures Accelerator Data and Analytics Platform (RDCA-DAP). Cure Mito and C-path are partnering to use the platform to aggregate data from other mitochondrial disease databases to expedite therapy development and facilitate clinical trials. An additional talk about data sharing was provided by Shannon Rego O’Rourke, research director of the Allstripes platform - a platform dedicated to supporting the drug development process by collecting and analyzing medical records on behalf of enrolled patients.

Parents had an opportunity to speak during a caregiver panel. They often expressed feeling alone in caring for their child. They shared that their sentiments stemmed from the lack of a cure for Leigh syndrome, in addition to the inaccessibility of information and experts in mitochondrial disease. During the symposium, we heard from Dr. Rachel Kramer, a clinical psychologist, who acknowledged the challenges of caregivers but also emphasized the importance of self-care and shared ways to maintain one’s well-being while caring for children with progressive diseases.

### Provider-patient interactions and rapport building

The symposium was a powerful venue for the exchange of the direct needs between families, healthcare providers, and scientists. Healthcare and research professionals infrequently have the opportunity to hear from patients and their loved ones. A direct result of the interactions between families, providers, and scientists during this symposium was the highlighted need for enhanced provider sensitivity training and effective communication between providers and researchers, and families.

While outcomes in research and clinical settings are often communicated as factual and objective, patients with mitochondrial disease and their families need sensitivity and compassion in the communication of poor prognoses. While families are certainly aware of the limited options available, providers need to remember to ask and understand what is important and meaningful to a given patient or family and make room for conversations on available clinical trials, facilitate community connections, and discuss ways to achieve the quality of life measures that are important for the patient and family in the time that they will have together.

The families in our symposium further highlighted how much healthcare providers and scientists can learn from them regarding the disease progression. As many mitochondrial diseases progress heterogeneously, a family member or caregiver is likely to be the expert in the room on the natural history of the given patient’s disease. As such, qualitative insight can be gained into the disease course through extensive history-taking and follow-up with families and caregivers. The conversational back-and-forth between family, provider, and scientists will likely underlie the success of future therapeutic investigations.

## Conclusion

The Cure Mito/Boston University iCAMP Leigh syndrome symposium was successful in highlighting several points. First, recent advances in gene editing technologies and mitochondrial disease models have facilitated, and will likely accelerate, novel insight into disease mechanisms. Second, clinical trials with small numbers of patients are beginning to identify therapies that improve outcomes in select patients with mitochondrial diseases with similar phenotypes. Third, the Leigh syndrome global patient registry continues to develop and grow, and will likely facilitate a better understanding of the prevalence of the different symptoms and disease progression. Most importantly, this symposium was successful with a turnout of over 200 participants from 34 countries in attendance, demonstrating the power of the community coming together and laying the groundwork for much more to come. Together, the advances presented, and the push for collaboration between all stakeholders, will hopefully result in novel treatment options for patients with Leigh syndrome and bring us into the future of mitochondrial disease management.

## Data Availability

Video recordings of all of the talks at the symposium are publicly and freely available on YouTube- https://www.youtube.com/playlist?list=PLfcCshCQuODdZdrLLF9sfAhQWfi8EJC-K.

## List of abbreviations

CDISC: Clinical Data Interchange Standards Consortium
CRISPR: Clustered, Regularly Interspaced Short Palindromic Repeats
DNA: deoxyribonucleic acid
FDA: Food and Drug Administration
iCAMP: integrative Cardiac Metabolism and Pathophysiology Program
iPSC: Induced Pluripotent Stem Cell
